# Endothelial Nitric Oxide Synthase Knockdown in Human Stem Cells Impacts Mitochondrial Biogenesis and Adipogenesis: Live-Cell Real-Time Fluorescence Imaging

**DOI:** 10.3390/jcm10040631

**Published:** 2021-02-07

**Authors:** Sylvia Lee-Huang, Philip Lin Huang, Paul Lee Huang

**Affiliations:** 1 Department of Biochemistry and Molecular Pharmacology, NYU Grossman School of Medicine, New York University, New York, NY 10016, USA; 2 Department of Medicine, Harvard Medical School and Massachusetts General Hospital, Boston, MA 02114, USA; huangp@helix.mgh.harvard.edu (P.L.H.); phuang1@partners.org (P.L.H.)

**Keywords:** endothelial nitric oxide synthase (eNOS), nitric oxide (NO), human mesenchymal stem cell (hMSC), mitochondrial biogenesis, adipogenesis, mitochondrial remodeling, live-cell real-time fluorescence imaging, molecular diagnostic medicine

## Abstract

We carried out live-cell real-time fluorescence imaging to follow the effects of genetic (siRNA) knockdown (KD) of endothelial nitric oxide synthase (eNOS) on mitochondrial biogenesis and adipogenesis in human mesenchymal stem cells (hMSCs). We report here that eNOS KD in hMSCs blocks mitochondrial biogenesis and adipogenesis. The transfer of mitochondria from normal hMSCs to eNOS-deficient hMSCs restores adipogenesis. Furthermore, cell-free mitochondria purified from normal hMSCs also restores adipogenesis in eNOS-deficient cells. Thus, eNOS and NO signaling are essential for mitochondrial biogenesis, and mitochondrial activity is indispensable for adipogenesis in hMSC differentiation. We mapped the path and identified the mechanisms of mitochondrial transfer. We captured real-time images of differentiated mature adipocytes in mitosis and replication. These results reveal that human stem cell-differentiated fat cells are capable of replication. This new finding offers novel insights into our understanding of fat cell expansion and the development of obesity. Real-time imaging in live cells allows synchronized investigation of mitochondrial biogenesis and adipogenesis in stem cell differentiation without reducing living cells to nonliving samples for functional analysis. Live-cell real-time imaging can thus be a faithful and immediate tool for molecular diagnostic medicine. Furthermore, our results suggest that mitochondrial remodeling can be a useful approach in treating adiposity, diabetes, and abnormalities in energy metabolism and vascular signaling.

## 1. Introduction

Endothelial nitric oxide synthase (eNOS)-derived nitric oxide (NO) regulates many important functions, including vascular tone and regional blood flow, vascular smooth muscle cell proliferation, leukocyte–endothelial interactions, and thrombosis [1,2,3,4,5]. The effect of eNOS-derived NO on mitochondria and its links to insulin resistance and obesity have been mostly studied in animal models [6,7,8,9,10]. Few studies have been performed in human stem cells. We reported that eNOS-derived NO plays distinct and separable roles in white and brown adipogenesis in human stem cells [11]. In brown adipocytes, eNOS-derived NO upregulates the expression of the thermogenic genes. In white adipocytes, eNOS-derived NO is required for mitochondrial biogenesis and adipocyte differentiation [11]. 

The aim of this study is to investigate the impact of eNOS knockdown (KD) in human stem cells on mitochondrial biogenesis and adipogenesis using live-cell real-time fluorescence imaging. We chose four read-outs to simultaneously assess gene transfer (green fluorescence protein), functional mitochondrial membrane potential (MitoTracker Red CMX Ros), cellular nuclei (Hoechst 33342), and cellular morphology (phase contrast) in living cells throughout the process of differentiation. Our results show that the genetic KD of eNOS in human mesenchymal stem cells (hMSCs) affects mitochondrial biogenesis and adipogenesis. The transfer of mitochondria from normal hMSCs to eNOS-deficient hMSCs restores adipogenesis. Moreover, cell-free mitochondria purified from normal hMSCs are equally effective in restoring adipogenesis in the eNOS-deficient hMSCs. These results confirm that eNOS and NO signaling are essential for mitochondrial biogenesis and that mitochondrial activity is essential for adipogenesis in human stem cells. Thus, eNOS and NO are important targets for the modulation of mitochondrial functions and adiposity. 

Mitochondria play critical roles in human health and diseases. In addition to providing energy for life functions, mitochondria serve as platforms and regulators for many cellular signaling pathways [12,13]. In addition to cardiovascular and metabolic diseases, mitochondrial dysfunction is associated with a wide range of other illnesses, including cancer and neurodegenerative diseases [14,15]. Intercellular transfer of mitochondria has been proposed as an attractive approach for organelle-based therapy [14,15,16,17,18,19]. The uptake of healthy mitochondria and subsequent functional recovery of recipient cells have been reported [14,15,16,17]. Our results demonstrate that live-cell real-time imaging can be used in the direct and immediate monitoring of mitochondria and their link to human stem cell adipogenesis. 

## 2. Experimental Section

### 2.1. Human Mesenchymal Stem Cell (hMSC)

Adipose-derived hMSCs were obtained from Thermo Fisher Scientific (Cat#SV3010201, Waltham, MA, USA). 

### 2.2. Genetic Knockdown of eNOS and Generation of eNOS-Deficient hMSCs 

Genetic KD of eNOS in hMSCs was carried out using Dharmacon SMARTvector Inducible Lentiviral eNOS siRNA vector (Human NOS3 4846, Dharmacon, Lafayette, CO, USA, Thermo Fisher Scientific, Waltham, MA, USA), according to the manufacturer’s instructions. This vector platform includes a Turbo-GFP (green fluorescence protein) reporter for visual tracking of transduction and expression upon induction and a puromycin resistance gene for the selection of transduced cells and the development of stable cell lines. The eNOS KD stable cell line that we developed in this study is designated eNOS-KD-hMSC. For KD control, a nontargeting (NT) vector was used. This NT vector platform also contains a Turbo-GFP reporter for visual tracking and a puromycin resistance gene for selection. The stable cell line of this nontargeting control is designated eNOS-NT-hMSC. 

### 2.3. Western Blot Analysis 

eNOS KD was confirmed by Western blot analysis of eNOS protein expression. Cells from the control hMSCs, eNOS-KD-hMSCs, and eNOS-NT-hMSCs were lysed on ice by ultrasonication for 10 min with lysis buffer (8 M urea, 50 mM IAA (iodoacetamide), 10 mM DTT (dithiothreitol), and protease inhibitor). The lysate was centrifuged at 12,000 rpm at 4 °C for 10 min. The supernatant was collected, and the protein concentration was determined. Protein extracts (50 µg) from each of these cells were electrophoresed through a 10% SDS (sodium dodecyl sulfate) polyacrylamide gel and transferred onto a 0.22 μm nitrocellulose membrane. The blot was incubated with primary antibodies directed against eNOS (#ab76198, Mouse monoclonal, Abcam, Cambridge, MA, USA) overnight at 4 °C in a buffer containing 150 mM NaCl, 50 mM Tris-HCl, 0.05% Tween-20, and 5% powdered milk, pH 7.4. After washing five times for 5 min, it was incubated with a secondary antibody (ab205719, Goat Anti-Mouse IgG H&L (HRP)) for 1 h at room temperature. eNOS protein was identified by enhanced chemiluminescent (ECL) development solution (ab133406). GAPDH was used as an internal control. Anti-GAPDH (ab8245) was from Abcam. 

PGC-1α is a marker of mitochondrial biogenesis [20]. The impact of eNOS KD on the expression of PGC-1α protein was measured by Western blot analysis. Rabbit polyclonal PGC-1α (ab191838, Abcam) was used as the primary antibody. The secondary antibody was goat anti-rabbit IgG H&L HRP (ab205718).

### 2.4. Cell-Free Mitochondria Isolation and Purification

Cell-free mitochondria were isolated and purified from control hMSCs using the Qproteome Mitochondria Isolation Kit from QIAgen (Cat No. 37612. Germantown, MD, USA), following the manufacturer’s protocol. All steps were carried out at 4 °C. Briefly, cell suspension containing 2 × 10^7^ cells was transferred into a 15 mL conical centrifuge tube and centrifuged at 500× *g* for 10 min. The supernatant was removed and discarded. The cell pellet was washed with 1 mL of 0.9% sodium chloride solution. The cell pellet was resuspended in 2 mL ice-cold lysis buffer containing a protease inhibitor. The cell suspension was shaken gently on an end-over-end shaker for 10 min to ensure complete lysis of the cells. The lysate was centrifuged at 1000× *g* for 10 min. The supernatant was removed, and the cell pellet was resuspended in 1.5 mL ice-cold disruption buffer. Complete cell disruption was achieved by using a blunt-ended needle and a syringe, drawing the lysate slowly into the syringe and ejecting 10 times.

The lysate was centrifuged at 1000× *g* for 10 min. The supernatant contained mitochondria, and the pellet contained cell debris. The supernatant was transferred to a 1.5 mL centrifuge tube and centrifuged at 6000× *g* for 10 min. The supernatant containing the microsomal fraction was removed. The mitochondrial pellet was washed with 1 mL mitochondria storage buffer and centrifuged at 6000× *g* for 20 min. 

For high purity, the mitochondrial preparation was further purified by differential density gradient centrifugation. The mitochondrial pellet was resuspended in 750 μL of mitochondria purification buffer and layered onto a 2 mL microcentrifuge tube that contained 500 μL of disruption buffer under 750 μL of mitochondria purification buffer, centrifuged at 14,000× *g* for 15 min. Due to their different viscosities, the disruption buffer and mitochondria purification buffer did not readily mix, allowing them to be layered. A band containing mitochondria was formed in the lower part of the tube. The band containing purified mitochondria was collected, and 1.5 mL of mitochondria storage buffer was added to the mitochondrial band. The mitochondrial suspension was centrifuged at 8000× *g* for 10 min. This step was repeated three times until the mitochondria formed a pellet at the bottom of the tube. Finally, the purified mitochondrial pellet was resuspended in the mitochondria storage buffer for further analysis and use. From 2 × 10^7^ control hMSCs, about 60 µg of highly purified cell-free intact mitochondria was obtained. We quantitated this to determine the amount of purified mitochondria equivalent to the number of cells used in co-culture experiments. The cell-free mitochondria (C-F mitochondria) were characterized by MitoTracker Red CMXRos staining, mitochondrial protein analysis, and mitochondrial DNA analysis. The purified cell-free intact mitochondria were free from genomic and cytosolic contaminants. They were used for the restoration of adipogenesis in eNOS-deficient hMSCs.

### 2.5. Mitochondrial DNA (mtDNA) Preparation and Analysis

Mitochondrial DNA was isolated from purified mitochondria using a Mitochondrial DNA Isolation Kit (Cat #K280-50) from BioVision (Milpitas, CA, USA), following the manufacturer’s protocol. Mitochondrial DNA was analyzed by agarose gel (1%) electrophoresis of BamH1 digests. mtDNA was stained with ethidium bromide, viewed, and documented with a UV transilluminator. 

### 2.6. Cell Culture and hMSC Adipogenic Differentiation

The hMSCs were cultured in complete hMSC expansion medium (HyClone SH30875.KT, Northbrook, IL, USA) at 37 °C, 5% CO_2_, in a H_2_O incubator. Adipogenic differentiation was carried out in an adipogenic medium (HyClone SH30876.KT) containing insulin, IBMX (3-isobutyl-1-methylxanthine), and dexamethasone. The culture media were replaced with fresh media every 3 days. hMSC differentiation and adipogenesis were monitored in live cells and in real-time by fluorescence imaging. Lipid droplet formation and accumulation were visualized and recorded. Adipogenesis was confirmed by Oil Red O assay (Thermo Fisher Scientific Inc., Waltham, MA, USA) and by RT-PCR on the expression of adipogenic genes. 

### 2.7. RNA Isolation and Real-Time PCR

Total RNA was isolated from cells during differentiation, using TriPure Isolation Reagent (Roche Diagnostic, Basel, Switzerland), following the manufacturer’s instructions. Genomic DNA was removed from isolated RNA with DNase (M610A, Promega, Madison, WI, USA) according to the manufacturer’s protocol. The concentration and purity of the RNA samples were determined by NanoDrop spectrophotometer (Thermo Scientific, Waltham, MA, USA). Complementary DNA (cDNA) was produced from 1 μg of RNA using Taq-Man Reverse Transcriptase Reagents (Applied Biosystems, Waltham, MA, USA) according to the manufacturer’s instructions. To confirm adipogenesis, the expression of adipogenic/lipogenic genes was profiled, including transcription factor peroxisome proliferator activated receptor γ2 (PPARγ2), lipoprotein lipase (LPL), and lipid binding protein (αP2). 28S ribosomal RNA was used as the internal control. The primer sequences are provided in Table 1 [21].

### 2.8. Cell Labeling and Co-Culture

hMSCs were fluorescently labeled with MitoTracker Red CMXRos (M7512, Molecular Probes, Eugene, OR, USA) to stain the mitochondria red and with Hoechst 33342 (Molecular Probes) to stain the nuclei (DNA) blue. Labeled hMSCs were washed 3 times with complete medium to remove any free dye. The eNOS-KD-hMSCs were green due to the expression of green fluorescent protein (GFP). The cells were cultured and imaged directly on a glass bottom 96-well plate (MatTek, Ashland, MA, USA). 

For co-cultures, washed eNOS-KD-hMSCs were suspended in complete medium at 1 × 10^5^ cells/mL; 100 µL of this cell suspension, containing 10,000 cells, was plated into each well; and 100 µL of washed control hMSCs at a density of 1 × 10^3^ cells/mL, containing 100 cells, were then added to each well to give a ratio of eNOS-KD-hMSCs to control hMSCs of 100:1.

For restoration of adipogenesis in eNOS-deficient hMSC cultures using cell-free mitochondria, we used 1–2 ng of cell-free mitochondria and 1 × 10^4^ eNOS-deficient hMSCs. This amount of cell-free mitochondria and culture density were chosen to replicate the co-culture experiments. 

### 2.9. Live-Cell Real-Time Imaging

Live-cell real-time images were collected at 37 °C, 5% CO_2_ using a microplate incubator, with precision temperature, humidity, and CO_2_ controls, mounted on the microscope stage of a Leica inverted microscope equipped with an automatic power stage and an AF6000 camera. The PH (phase contrast), RFP (red, MitoTracker), DAP (blue, Hoechst 33342), and GFP (green, GFP) channels were used for imaging. Image analysis was performed using LCSAF software (Leica).

### 2.10. Replication of Results

For all live-cell, real-time fluorescence studies, independent experiments were repeated at least three times for each experiment.

## 3. Results

### 3.1. Confirmation of eNOS Knockdown in hMSCs

**Figure 1:**
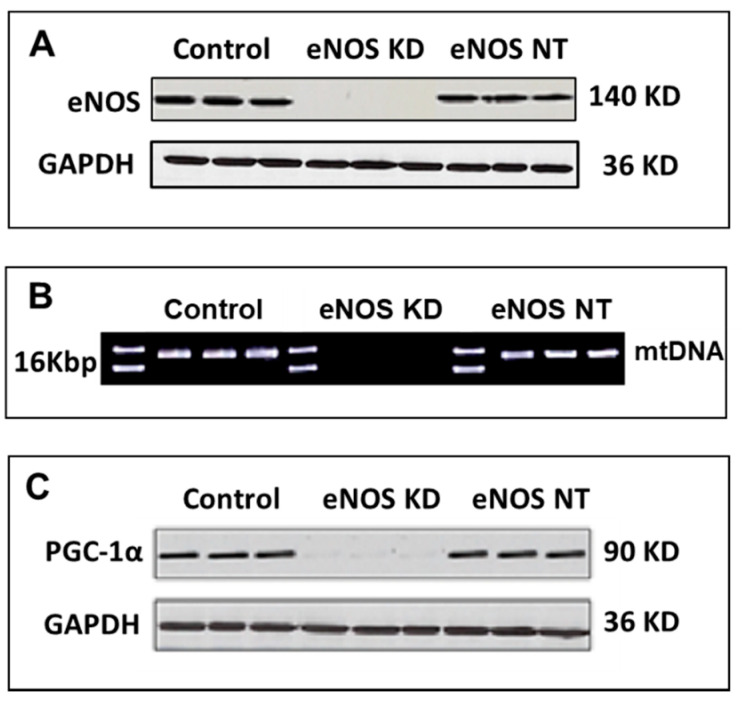
Confirmation of endothelial nitric oxide synthase (eNOS) knockdown (KD) in human mesenchymal stem cells (hMSC): (**A**) Western blot analysis of eNOS protein expression, (**B**) mitochondria DNA (mtDNA) analysis by agarose gel electrophoresis, and (**C**) Western blot analysis of PGC-1α protein expression. Abbreviations: KD, knockdown; NT, nontargeting control.

To confirm eNOS knockdown, we carried out Western blot analysis on the expression of the eNOS protein in control hMSCs, eNOS-KD-hMSCs, and eNOS-NT-hMSCs. Figure 1A shows the results of the Western blot analysis on the expression of the eNOS protein in these cells. The eNOS protein was clearly seen in the control hMSCs and in the eNOS-NT-hMSCs, but the eNOS protein was not detected in the Western blot of the eNOS-KD-hMSCs.

### 3.2. Live-Cell Real-Time Fluorescence Imaging Showing eNOS KD in hMSCs Impacts Mitochondrial Biogenesis and/or Function

To observe the impact of eNOS KD in hMSCs on mitochondrial biogenesis and function in live-cells real-time, we used fluorescence imaging using MitoTracker Red CMXRos staining. This red-fluorescent dye stains only intact mitochondria in live cells, and its accumulation depends on the functional mitochondrial membrane potential [22,23,24]. Thus, the presence of the red stained cells reflects not only the presence of mitochondria in these cells but also their intact function. 

**Figure 2:**
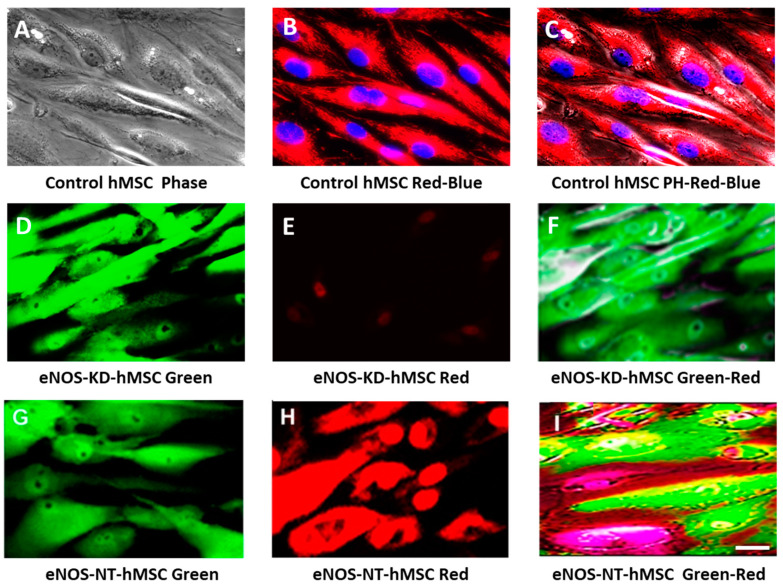
Live-cell real-time fluorescence images showing the effect of eNOS KD on mitochondrial biogenesis and/or function in hMSC: (**A**–**C**) control hMSCs in phase (PH), red-blue, and PH-red-blue overlay; (**D**–**F**) eNOS-KD-hMSCs in green, red, and green-red overlay; and (**G**–**I**) eNOS-NT-hMSCs in green, red, and green-red overlay. Scale bar 10 µm.

Figure 2 consists of groups of live-cell real-time fluorescence images of control hMSCs, eNOS-KD-hMSCs, and eNOS-NT-hMSCs, showing the effect of eNOS knockdown on mitochondrial biogenesis, mitochondrial function, or both. Frames A–C are live-cell real-time fluorescence images of control hMSCs. Frame A is viewed under phase contrast, illustrating the structure and morphology of normal control hMSCs. Frames B and C are control hMSCs fluorescently labeled with MitoTracker Red CMXRos and with Hoechst 33342 to stain the mitochondria red and the nuclei (DNA) blue, viewed under red (RFP)-blue (DAP) and red (RFP)-blue (DAP)-phase contrast (phase) overlay. These images demonstrate that control hMSCs with normal mitochondrial biogenesis and intact mitochondrial function are well-stained with MitoTracker Red CMXRos. 

Frames D–F are live-cell real-time fluorescence images of eNOS-KD-hMSCs in green (GFP), red (RFP), and green (GFP)-red (RFP) overlay. In frame D, the expression of green fluorescence protein in the targeting vector renders the cells green. In frame E, only a few ill-stained red spots can be seen. In frame F, no well-stained (red) mitochondria were detected in the green cells. This result indicates that eNOS KD impacts mitochondrial biogenesis, mitochondrial function, or both.

Frames G–I show the nontargeting control eNOS-NT-hMSCs in green (GFP), red (RFP), and green (GFP)-red (RFP) overlay. In frame G, the eNOS-NT-hMSCs are green because the NT vector contains the green fluorescence protein reporter gene for selection. In frame H, the cells were well-stained with MitoTracker Red CMXRos, indicating normal mitochondrial biogenesis and mitochondrial function in these cells. In frame I, the red mitochondria in the green cells are clearly seen as yellow and orange dots/areas. eNOS expression was unaffected, and there was no impact on mitochondrial biogenesis/function. These results show that the knockdown process, the insertion, and the expression of the green fluorescence protein reporter gene in the hMSCs had no effect on mitochondrial biogenesis and function. 

### 3.3. Confirmation on Impact of eNOS Knockdown on Mitochondrial Biogenesis

To confirm the impact of mitochondrial biogenesis in the eNOS KD cells, mtDNA was isolated and purified from control hMSCs, eNOS-KD-hMSCs, and eNOS-NT-hMSCs; was digested with BamHI; and was analyzed by agarose gel electrophoresis. BamH1 specifically recognizes GGATCC and cuts at the position of G/G. There is only one BamHI restriction site in mtDNA at nt14,258–nt14,263. Thus, upon BamHI treatment, the loop of mtDNA opens and forms linear mtDNA of 16–17 Kbp for unambiguous identification. Figure 1B shows the results of the mtDNA analysis. Intact mtDNA was clearly detected in both control hMSCs and in eNOS-NT-hMSCs, but it was not detected in eNOS-KD-hMSCs. 

PGC-1α is a transcription factor involved in mitochondrial biogenesis [20]. We thus investigated the effect of eNOS knockdown on the expression of PGC-1α protein by Western blot analysis. Figure 1C shows the results of the Western blot analysis of PGC-1α protein expression. PGC-1α protein was visibly present in both control hMSCs and eNOS-NT-hMSCs but was barely detectable in eNOS-KD-hMSCs. This indicates that the mechanism by which eNOS gene knockdown affects mitochondria is upstream of PGC-1α, confirming its effect on mitochondrial biogenesis.

### 3.4. eNOS KD in hMSCs Impacts Adipogenesis, and Transfer of Healthy Mitochondria from Control hMSCs to eNOS-Deficient hMSCs Restores Adipogenesis

**Figure 3:**
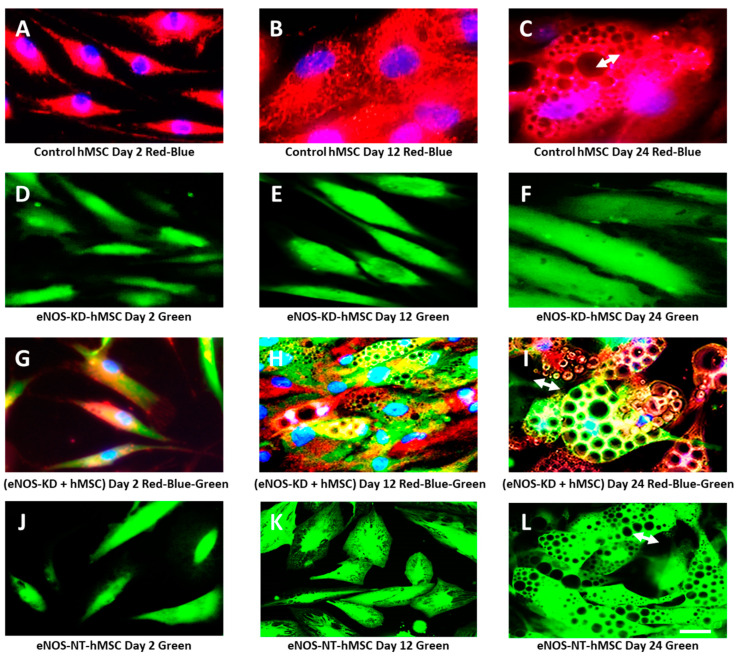
Live-cell real-time fluorescence images showing the effects of eNOS KD on adipogenesis and that mitochondria transfer from control hMSCs restores adipogenesis: (**A**–**C**) control hMSCs on days 2, 12, and 24 (red-blue overlay); (**D**–**F**) eNOS-KD-hMSCs on days 2, 12, and 24 (green); (**G**–**I**) co-cultures of eNOS-KD-hMSCs and control hMSCs on days 2, 12, and 24 (red-blue-green overlay); and (**J**–**L**) eNOS-NT-hMSCs on days 2, 12, and 24 (green).

Figure 3 shows the impact of eNOS knockdown on adipogenesis in hMSCs and the restoration of adipogenesis in eNOS-deficient hMSCs by the transfer of healthy mitochondria from normal hMSCs in co-culture, as monitored by live-cell real-time fluorescence imaging during the course of adipogenic differentiation. Adipogenesis was followed by the formation and accumulation of lipid droplets (LD) and lipid bodies (LB). LD and LB are highly reflective. Under fluorescence microscopy, they are seen as distinctive black round vacuole-like organelles in the cells. 

Figure 3A–C are live-cell real-time fluorescence images of control hMSCs cultured in adipogenic media on days 2, 12, and 24, respectively. The cells were stained with MitoTracker Red CMXRos and Hoechst 33342 (mitochondria red and nucleus blue). Control hMSCs with healthy mitochondria showed normal adipogenesis. Small lipid droplets started to appear between days 6 to 8. By day 12, a significant accumulation of lipid droplets occurred. Adipogenesis peaked by days 20–24, with the appearance of large LD (white arrows). At this time, mature fat cells are also seen. 

Figure 3D–F are live-cell real-time fluorescence images of eNOS-KD-hMSCs cultured in adipogenic media on days 2, 12, and 24. No lipid droplets were detected in these eNOS-deficient cells, indicating that eNOS KD impaired mitochondrial biogenesis and/or function, which in turn impacted adipogenesis. 

Figure 3G–I show co-cultures of eNOS-KD-hMSCs and control hMSCs. On day 2, as shown in frame G, healthy mitochondria (red) from control hMSCs entered the (green) eNOS-KD-hMSCs and are seen as yellow/orange dots (red in green). By day 12 (frame H), restoration of adipogenesis in the eNOS-deficient hMSCs became apparent. Lipid droplet formation and accumulation are clearly visible. By day 24 (frame I), large lipid droplets seen as vacuole-like organelles (white arrows, large reflective black areas) were present.

Figure 3J–L show eNOS-NT-hMSCs, which are nontargeted controls. They contained normal and functional mitochondria and showed normal adipogenesis. Tiny lipid droplets began to emerge between 6 to 8 days. By day 12, there was substantial accumulation of various sizes of lipid droplets. By day 24, large lipid bodies seen as large vacuole-like organelles (white arrows, black areas) were present in all the cells. 

### 3.5. Confirmation on the Impact of eNOS Knockdown on Adipogenesis

**Figure 4:**
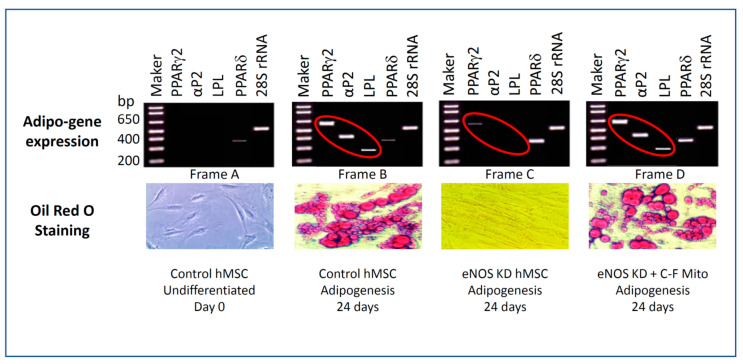
Confirmation of adipogenesis by RT-PCR profiling on the expression of adipogenic genes and Oil Red O staining of lipid droplets.

Figure 4 shows confirmation of the impact of eNOS knockdown on adipogenesis. Lipid formation and accumulation were verified by Oil Red O staining. The expression of adipogenic genes was confirmed by RT-PCR. 

Oil Red O is a lipophilic dye that stains lipid droplets in fat cells formed during adipogenesis to red. As seen in Figure 4, bottom row, undifferentiated hMSCs on day 0 contain no lipid droplets and do not stain with Oil Red O. Control hMSCs with normal mitochondrial biogenesis, cultured in adipogenic medium for 24 days, show normal adipogenesis, lipid droplets, and lipid bodies, and the cells stain with Oil Red O dye. eNOS-KD-hMSCs cultured in adipogenic media for 24 days showed no adipogenesis, no lipid droplet formation, and no staining with Oil Red O. eNOS-KD-hMSCs cultured with cell-free mitochondria were purified from normal hMSCs in an adipogenic medium for 24 days. The cell-free mitochondria (C-F Mito) from normal hMSCs restored adipogenesis in the eNOS-deficient cells. Lipid droplets formed in these developed adipocytes, and they stained red with Oil Red O. 

To confirm the impact of eNOS KD on gene expression during adipogenesis, we carried out RT-PCR. Table 1 shows the PCR primers of the genes we examined. We compared the expression profile of adipogenic genes [25,26] including transcription factor peroxisome proliferator activated receptor γ2 (PPARγ2), lipoprotein lipase (LPL) and lipid binding protein (αP2) in undifferentiated hMSCs, differentiated control hMSCs, eNOS-KD-hMSCs in adipogenic medium for 24 days, and eNOS-KD-hMSCs cultured with cell-free mitochondria purified from control hMSCs in adipogenic medium for 24 days. PPARγ2 is essential in driving the differentiation of hMSCs to pre-adipocytes. LPL and αP2 are markers of adipocytes and play major roles in the metabolism and transport of lipids in fat cells. We also profiled the expression of PPARδ, which is known to be antiadipogenic. 28S RNA was used as the internal control.

As seen in Figure 4, top row, undifferentiated control hMSCs showed no expression of adipogenic genes. Control hMSCs show expression of adipogenic genes PPARγ2, LPL, and αP2. eNOS KD in hMSCs blocks adipogenesis and the expression of the adipogenic genes PPARγ2, LPL, and αP2. In the presence of cell-free mitochondria purified from control hMSCs, uptake of the control mitochondria by the eNOS-deficient hMSCs restored adipogenesis. The expression of the adipogenic genes PPARγ2, LPL, and αP2 becomes evident. The expression of the internal control 28S rRNA remained constant in all of these cells. These results confirm that eNOS KD in hMSCs blocks adipogenesis and adipogenic gene expression. Interestingly, our results also indicate that eNOS KD upregulates the expression of PPARδ, the antiadipogenic gene, as well.

### 3.6. Mitochondrial Transfer, Adipogenesis Restoration, and Adipocytes Replication

**Figure 5:**
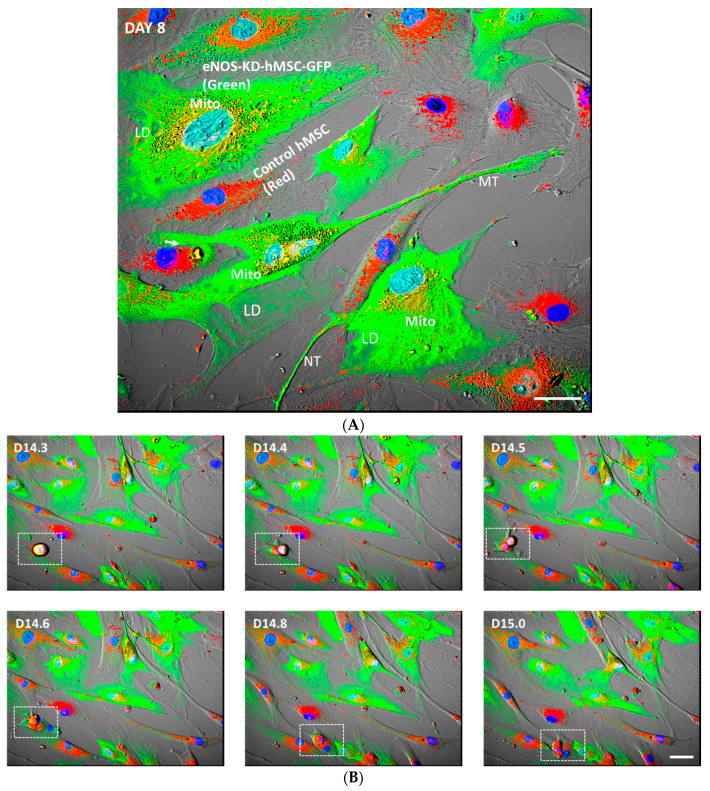

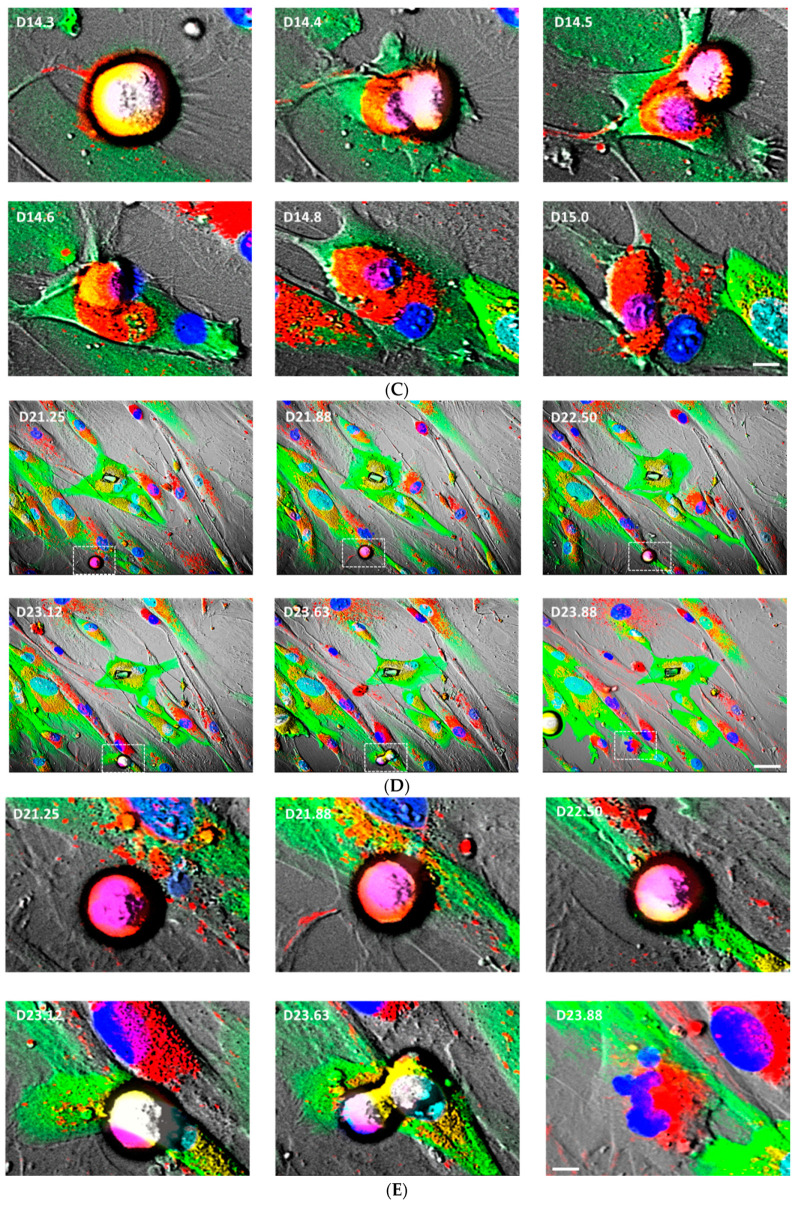
(**A**) Co-culture of eNOS-KD-hMSCs (green) and control hMSCs (red) on day 8 showing the transfer of mitochondria (red) and the restoration of adipogenesis in the eNOS-KD-hMSCs. Scale bar 10 µm. (**B**) Adipogenesis and adipocyte replication in co-culture on days 14 to 15. (**C**) Close up of white dotted rectangular areas in (**B**), days 14–15. (**D**) Continued adipogenesis and adipocyte replication on days 23 to 24. Scale bar, 10 µm. (**E**) Close up of white dotted rectangular areas in (**D**), days 23–24.

To better characterize mitochondrial transfer, we looked for known pathways by which mitochondria are known to transfer between cells. Figure 5A shows the live-cell real-time fluorescence image of a co-culture of eNOS-KD-hMSCs and control hMSCs in an adipogenic medium on day 8. This image shows the transfer of mitochondria (red) from the control hMSCs (red cells) to the eNOS-deficient cells, eNOS-KD-hMSCs (green cells). The red mitochondria were transferred from the control hMSCs to the eNOS-deficient cells (green cells) by direct cell-to-cell contact, through nanotubes (NT) and microtubes (MT). The red mitochondria in the green cells are seen as orange and/or yellow dots and/or areas. The restoration of adipogenesis in the eNOS-deficient hMSCs was clearly demonstrated by the formation and accumulation of lipid droplets (LD) in the green cells (Supporting Video S1). 

Figure 5B consists of time lapse fluorescence images of the co-culture between days 14 to 15. These pictures recorded images of the moment that a differentiated mature adipocyte (marked by the white dotted line) on day 14.3 entered mitosis through days 14.4 to 14.8 and finally divided into two lipid droplets containing daughter cells (marked by the white dotted line) on day 15. Figure 5C is the close-up of the time lapse images of the area marked by the white dotted line in Figure 5B. These images reveal the key stages of a dividing fat cell. The lipid body is seen as the reflective black area in the cells (white arrows).

Figure 5D shows time lapse fluorescence images of the same co-culture between days 21 and 24. The areas marked by the white dotted line show another differentiated mature adipocyte undergoing mitosis. Figure 5E shows close-up times lapse images of the adipocyte in the areas marked by the white dotted line Figure 5D. These fascinating live-cell real-time images faithfully document the division of yet another mature fat cell into two lipid droplet-containing daughter cells. 

It is not known whether differentiated lipid-filled mature adipocytes are capable of replication. This is a critical issue that is central to our understanding of lipid metabolism, fat expansion, and obesity. Generally, it is believed that differentiated mature adipocytes with accumulated lipid droplets exit the normal cell cycle and stop mitosis [27]. However, it has also been reported that differentiated 3T3-L1 adipocytes containing lipid droplets were able to divide [28]. In our study here, we captured, in real time, the moments when differentiated mature adipocytes from human stem cells underwent mitosis and divided into two daughter cells. These results show that human stem cell-differentiated fat cells are capable of division. This new finding offers novel insights in our understandings on fat cell expansion and the development of obesity.

### 3.7. Cell-Free Mitochondria Purified from Control hMSCs Are Capable of Restoring Adipogenesis in eNOS-Deficient hMSCs

**Figure 6:**
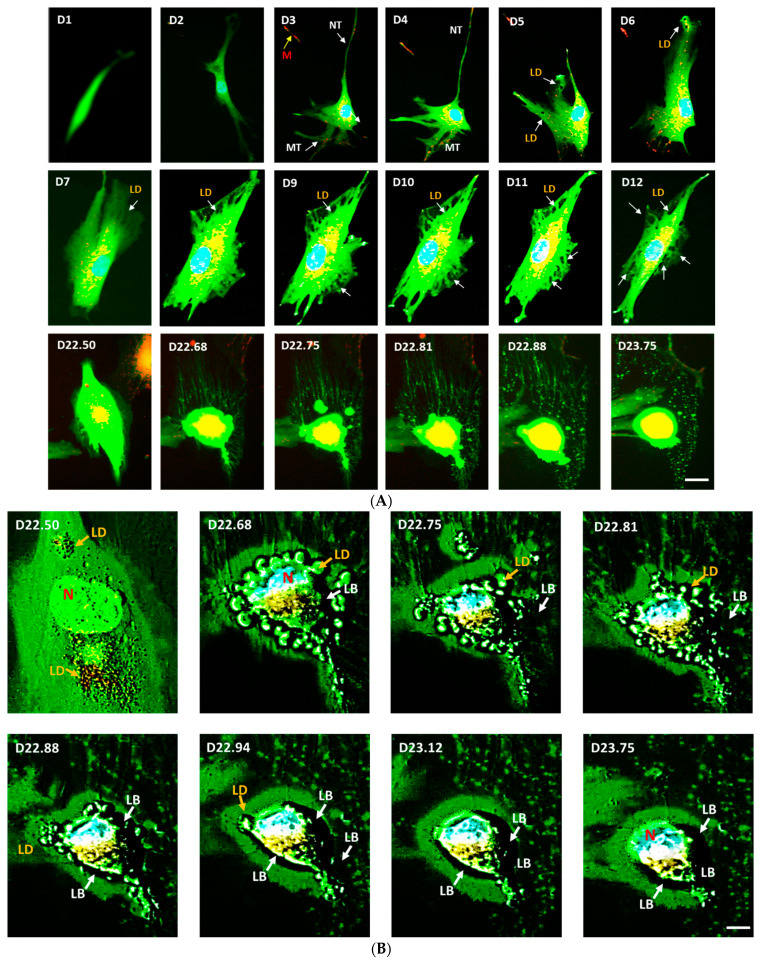
(**A**) Time-lapse fluorescence images of eNOS-KD-hMSCs cultured in the presence of cell-free mitochondria purified from control hMSCs from days 1 to 24: cell-free mitochondria purified from control hMSCs restore adipogenesis in eNOS-deficient hMSCs. Scale bar 10 µm. (**B**) Close-up time lapse fluorescence images of the culture in (**A**). LD: lipid droplets; LB: lipid bodies; D: day; N: nucleus.

To investigate whether purified cell-free mitochondria from control hMSCs are capable of restoring adipogenesis in eNOS-deficient hMSCs, we isolated and purified mitochondria from control hMSCs. The purified mitochondria, free from any cellular contaminants, were added to cultures of eNOS-deficient hMSCs. In this system, the only intact cells are the eNOS-deficient hMSCs in green. We followed the uptake of the cell-free red mitochondria by the eNOS-deficient green cells and the restoration of adipogenesis in live-cell real-time fluorescence imaging. These results are shown in Figure 6A,B and Supporting Videos S2 and S3. Our results demonstrate that purified cell-free mitochondria from control hMSCs are capable of restoring adipogenesis in eNOS-deficient hMSCs. 

Figure 6A represents the time lapse fluorescence images of eNOS-KD-hMSCs cultured in adipogenic medium in the presence of cell-free mitochondria purified from control hMSCs from days 1 to 24. We focused on a field showing a single eNOS-KD-deficient green cell. This series of live-cell real-time fluorescence images reveals that uptake of the purified cell-free healthy mitochondria restores adipogenesis in eNOS-deficient green cells. The cell-free red mitochondria, seen as red dots, enter the eNOS-deficient green cells, seen as yellow and orange dots and areas. On days 2 to 4, the cells started to undergo morphological transformation in coordination with adipogenic differentiation. The cells began to spread out with extended long nanotubes and short microtubes. These extended tubular structures were engaged in transporting the cell-free mitochondria. The cell-free red mitochondria, seen as orange dots in the green tubes (white arrows), travelled to the inside of the cell and reached the blue nucleus, seen as yellow areas. On days 5 and 6, the formation of small lipid droplets (LD) was visible (white arrows). By days 7 and 8, the accumulation of lipid droplets into large lipid bodies was visible. By days 9 to 12, the formation and accumulation of the lipid droplets and lipid bodies continued and extended around the entire cell. By days 22 to 24, the transformation and maturation of the differentiated lipid containing adipocyte into a mature fat cell was evident. 

Figure 6B shows close-up time lapse fluorescence images between days 22 and 24 of Figure 6A. Cellular transformations characteristic of adipocyte maturation are clearly visible during this period. On day 22.50, the cell was filled with large amounts of lipid droplets (LD) and lipid bodies (LB). On day 22.68, the cell was transformed from an elongated to spherical cell shape. In doing so, the cell nucleus (N, blue) was pushed to one end of the cell. In addition, many round lipid droplets (LD) were seen in the entire inner cellular space. From day 22.75 onward, as the lipid droplets fused into lipid bodies (LB), the LD started to disappear and the LB expanded consequently. Finally, by day 23.75, the fusion of LD into LB reached completion. At this point, the small LDs were fused into a large LB, filling the entire cell. These results indicate that restoration of adipogenesis in this eNOS-deficient hMSC by cell-free mitochondria from the control hMSC was accomplished. 

## 4. Discussion and Conclusions

In this study, we used live-cell real-time fluorescence imaging to investigate the impact of eNOS genetic (siRNA) knockdown in hMSCs on mitochondrial biogenesis and adipogenesis. This technique allows for continuous documentation of cellular events during the entire course of adipogenesis. It enabled us to track the paths of cell-to-cell mitochondria transfer, to map the phases of lipid droplet formation, to monitor the stages of adipocyte differentiation, and to capture the exact moment of fat cell replication. 

MitoTracker Red CMXRos is well suited for our multicolor labeling experiments since its red fluorescence is well resolved from the green fluorescence used to track gene targeting in eNOS-kD-hMSCs. MitoTracker Red CMXRos stains only intact mitochondria in live cells, and its accumulation depends on functional mitochondrial membrane potential. Thus, staining reflects not only the presence of mitochondria but also their intact function. We are able to simultaneously and continuously document, in live cells and in real time, mitochondrial transfer (red mitochondria to green cells as orange/yellow spots) and restoration of adipogenesis (lipid droplet formation, accumulation, and adipocytes maturation) during the entire process for 24 days. 

Our results show for the first time that genetic knockdown of eNOS in human stem cells blocks mitochondrial biogenesis and adipogenesis. These results indicate that eNOS and NO signaling are essential for mitochondrial biogenesis and that mitochondrial activity is indispensable for adipogenesis in human stem cells. Whether there are any additional energy sources for ATP production in eNOS-KD-hMSCs is not known. 

The restoration of adipogenesis by the transfer of mitochondria from control hMSCs to eNOS-deficient hMSCs as well as by cell-free mitochondria purified from healthy hMSCs demonstrate that human mitochondria can be transferred from one cell to another. We identified a variety of pathways for human mitochondrial transfer including by direct cell-to-cell contact, through thin nanotubes or large microtubes. Each of these mechanisms has been described [14,15,16,17,18]. High-resolution images from the co-culture experiments document evidence for each of these mechanisms in the hMSCs. 

The transfer of healthy mitochondria from control hMSCs to eNOS-deficient hMSCs restores adipogenesis in the eNOS-deficient cells, as evidenced by visualization in real-time of lipid droplet formation, accumulation, and maturation of fat cells. These results were confirmed by classical Oil Red O analysis of the lipid droplets in the differentiated adipocytes as well as by the expression of adipogenic genes by RT-PCR. 

Importantly, we captured in live-cell real-time the mitosis of the human stem cell-differentiated adipocyte and its divisions into two daughter fat cells. These results are significant to our understanding of fat cell expansion and the development of obesity in humans. Currently, most of what we know about fat cell replication has come from the results obtained of rodents either in cell culture or in an animal model. These are examples of adipose function that may not translate fully from the mouse to the human. Hence, knowledge on human adipocyte replication is critical to the control and management of human obesity. 

Finally, live-cell real-time fluorescence imaging allows direct visualization and instantaneous data documentation. It captures the interplay of cellular components in live cells without reducing living cells to nonliving samples for objective analysis that diverges from innate physiological conditions. Thus, live-cell real-time fluorescence imaging is a unique molecular diagnostic tool because it allows for direct simultaneous visualization of separate intracellular events relevant to health and disease (gene transfer, mitochondrial function/membrane potential, and differentiation) in living cells. 

## Figures and Tables

**Table 1:** PCR primers for adipogenic genes in human mesenchymal stem cell (hMSC) adipogenesis differentiation.

Gene	Primer Sequence (Sense/Antisense)	Product (bp)	Gene ID
Marker	1 kb plus DNA ladder with 200, 300, 400, 500, 650, 850, 1000 bp	200–1000	
PPARγ Adipogenic	5′-GGATGTCGTGTCTGTGGAGA-3′ 5′-TGAGGAGAGTTACTTGGTCG-3′	630	BC006811
LPL Adipogenic	5′-GAGATTTCTCTGTATGGCACC-3′ 5′-CTGCAAATGAGACACTTTCTC-3′	276	BC011353
αP2 Adipogenic	5′-GTACCTGGAAACTTGTCTCC-3′ 5′-GTTCAATGCGAACTTCAGTC-3′	418	BC007538
PPARδ Anti-adipogenic	5′-GGTGAATGGCCTGCCTCCCTACAA-3′ 5′-CACAGAATGATGGCCGCAATGAAT-3′	380	BC007578
28S RNA Internal control	5′-GTGCAGATCTTGGTGGTAGTAGC-3′ 5′-AGAGCCAATCCTTATCCCGAAGTT-3′	589	BC000380

PPAR: proliferator activated receptor; LPL lipoprotein lipase.

## Supplementary Materials

The following are available online at https://zenodo.org/record/4517434#.YCC6n3kRVPZ, Supporting Video S1: Co-culture of eNOS-KD-hMSC (green) and control hMSC (mito red, nucleus blue), Days 8 to 24; Supporting Video S2: eNOS-KD-hMSC (Green) cultured with purified cell-free mitochondria from control hMSC (Red), Day 1 to day 12; Supporting Video S3: eNOS-KD-hMSC (Green) cultured with purified cell-free mitochondria (Red) from control hMSC, Day 13 to day 24.
